# The fight against lymphatic filariasis: perceptions of community drug distributors during mass drug administration in coastal Kenya

**DOI:** 10.1186/s40249-020-0638-1

**Published:** 2020-03-02

**Authors:** Caroline Kusi, Peter Steinmann, Sonja Merten

**Affiliations:** 1 Swiss Tropical and Public Health Institute, Basel, Switzerland; 2 University of Basel, Basel, Switzerland

**Keywords:** Neglected tropical diseases, Lymphatic filariasis, Mass drug administration, Community drug distributors, Preventive chemotherapy, Qualitative methods, Community health, Health service

## Abstract

**Background:**

Lymphatic filariasis (LF), a neglected tropical disease (NTD) and leading cause of global disability, is endemic in 32 countries in Africa with almost 350 million people requiring regular drug administration, and only 16 countries achieving target coverage. Community Drug Distributors (CDDs) are critical for the success of NTD programs, and the distribution of medicines during mass drug administration (MDA) in Africa; however they could also be a weak link. The primary aim of this study is to explore and describe perceptions of CDDs during MDA for LF in Mvita sub-county in Mombasa county and Kaloleni sub-county in Kilifi county, Kenya; and provide recommendations for the effective engagement of communities and CDDs in low-resource settings.

**Methods:**

In September 2018, we conducted six focus group discussions with community members in each sub-county, three with men aged 18–30, 31–50, and 51 years and above and three with women stratified into the same age groups. In each sub-county, we also conducted semi-structured interviews with nine community health extension workers (CHEWs), the national LF focal point, the county NTD focal points, and seven community leaders. Content analysis of the data was conducted, involving a process of reading, coding, and displaying data in order to develop a codebook.

**Results:**

We found that several barriers and facilitators impact the engagement between CDDs and community members during MDA. These barriers include poor communication and trust between CDDs and communities; community distrust of the federal government; low community knowledge and perceived risk of LF, poor timing of MDA, fragmented supervision of CDDs during MDA; and CDD bias when distributing medicines. We also found that CDD motivation was a critical factor in their ability to successfully meet MDA targets. It was acknowledged that directly observed treatment and adequate health education were often not executed by CDDs. The involvement of community leaders as informal supervisors of CDDs and community members improves MDA.

**Conclusions:**

In order to achieve global targets around the elimination of LF, CDDs and communities must be effectively engaged by improving planning and implementation of MDA.

## Background

Neglected Tropical Diseases (NTDs) affect more than 2 billion people around the world, causing disability and death in vulnerable populations [1]. Thirty-two of the 52 countries that require preventive chemotherapy are in Africa, and almost 350 million people in Africa require regular drug administration. Only 16 countries in Africa achieved target coverage [2]. LF is one of five NTDs whose transmission cycle can be interrupted through annual large-scale distribution or mass drug administration (MDA) of preventive chemotherapy. In Africa, MDA campaigns usually rely on community drug distributors (CDDs).

Today, CDDs are critical for the success of many NTD programs, and the achievement of current global targets for the elimination of diseases like LF and onchocerciasis; however they could also be a weak link [3, 4]. Studies conducted in several African countries have shown the factors that jeopardize the sustainability and success of community-directed treatment programs, as well as the barriers and facilitators of coverage and compliance, including the motivation and performance of CDDs. A study profiling the best performing CDDs in Uganda demonstrated that treatment rates were associated with CDD characteristics rather than the willingness of community members to comply with treatment [5]. In some contexts, the selection and recruitment of CDDs are not led by the community or made transparent, resulting in community resistance and lack of trust of CDDs [6, 7]. A study conducted in Ethiopia found that CDDs had poor knowledge, attitude and practices related to onchocerciasis, leading to low participation of community members in MDA [8]. In Tanzania and Nigeria, researchers showed that CDDs did not understand the cause and transmission of the disease due to inadequate trainings. As a result, CDDs passed on wrong information to community members about LF, impacting community trust of CDDs [6, 9]. Also, CDDs in Northern Nigeria did not understand how to engage with individuals with physical disabilities and other complex conditions due to inconsistencies in messages during trainings [9].

Community drug distributors’ preferences for financial and material incentives, and their effect on motivation, are important factors in the implementation of MDA campaigns. In a study conducted in Uganda, Fleming and colleagues found that CDDs were driven by both intrinsic and extrinsic motivations. They were motivated to serve their communities and gain recognition; and also desired financial and material incentives, such as T-shirts, bags, hats, boots and waterproof coats with the program logo and certificates [10]. They also felt that financial compensation should reflect out of pocket expenses and opportunity costs incurred during MDA [10]. Opportunity costs can include missing out on farming and other small-scale ventures, as well as household food and school costs [10, 11]. In Ghana and Nigeria, CDDs preferred certificates, T-shirts, bicycles, ID cards, hats, preferential treatment at the district hospitals or health centres as compensation for their work [9, 11].

CDDs have reported that the support given by communities and the NTD program is not enough motivation [9, 10]. In Nigeria, Kenya and Ghana, CDDs expressed a desire to see their supervisors throughout MDA in order to increase their confidence and promote community acceptance of MDA [7, 9, 11]. The lack of supportive supervision of CDDs is often due to the high workload of supervisors during MDA campaigns and the provision of routine health services within healthcare centres [6, 11]. In Cameroon, CDDs working in the onchocerciasis program reported that low appreciation and community support affected their motivation [12]. Furthermore, in Nigeria, positive feedback from communities was a major motivating factor, resulting in feelings of happiness and fulfilment for CDDs. Also, supervisors and CDDs wanted more feedback and appreciation from the health sector, and to be acknowledged as contributors to population health [9].

In addition, CDDs’ workload during MDA also impacts their motivation and performance. In Tanzania, CDDs mentioned that they did not receive adequate time in order to achieve their performance goals [6]. In Nigeria, high attrition among CDDs resulted in high workload for the remaining CDDs, resulting in low motivation and disengagement [9]. In India and Ghana, CDDs reported difficulties with being able to deliver medicines in the time frame they were allotted [11, 13]. In efforts to achieve performance targets, CDDs are not compensated for the extra time they use to distribute medicines [6].

Furthermore, CDDs have reported a lack of resources and medicines to be able to perform well during MDA. In Uganda and Nigeria, researchers found that drug stock outs due to inaccurate census, insufficient transportation, bags to carry program documents, clean water for ingesting medicines, and insufficient quantities of registers and measuring sticks impacted the on-the-job experiences of CDDs [9, 10]. The inability to properly store and dispense tablets, and offer communities treatment for drug side effects also hinder CDDs’ work [10, 14].

Although studies have contributed to our understanding of the many challenges that demotivate and disengage CDDs and communities, additional evidence is needed to identify pathways for improving and sustaining the motivation of CDDs and engagement of communities. A systematic review of the factors influencing the motivation of CDDs concluded that there is a need for more research on measures that improve CDD motivation within the context of the local health system and changing sociocultural environment [15]. The investigation of the perceptions of CDDs can reveal the measures and pathways needed to better equip and engage CDDs.

### The Kenyan context

Evidence for the engagement of CDDs for the elimination of LF in Kenya is limited. Findings from studies conducted over five years ago in Kenya indicate that lack of supervision, lack of community trust in CDDs, inadequate training for CDDs and CDDs’ inability to reach households resulted in low coverage and compliance [7, 16]. Given the changing socio-cultural and economic landscape of countries like Kenya, and recent efforts to deliver multiple combinations of medicines to accelerate the elimination of LF, there are added pressures on CDDs to not only meet the demands of the NTD program, but also those of their families and communities. The NTD community needs a better understanding of how the roles, perceptions and job experiences of CDDs have changed in Kenya amidst new and intensified global, national, sub-national commitments and agendas. This new understanding can inform the development and implementation of recommendations, and ensure that no one is left behind.

## Methods

### Research design

This qualitative study used semi-structured key informant interviews and focus group discussions. Semi-structured interviews were conducted with community leaders, community health extension workers (CHEWs), and NTD program officials at the county and national levels. CHEWs are formally trained health care workers responsible for supervising CDDs during MDA. Focus group discussions were conducted with community members. The aim of these interviews was to explore the perception of CDDs during MDA.

### Setting

The study was conducted in September 2018 in coastal Kenya in Mvita sub-county, an urban area in Mombasa county, and Kaloleni sub-county in a rural area in Kilifi county. Two sites with coverage below the treatment threshold of greater than or equal to 65% in the two MDA rounds preceding this study were selected to compare differences and similarities in perceptions of CDDs. In 2015 and 2016, Kaloleni sub-county achieved coverage of 61 and 58% respectively, and in 2016 and 2017, Mvita sub-county achieved coverage of 51 and 59% respectively.

### Data collection

#### Focus groups

We conducted six focus group discussions with community members in each sub-county. Three focus groups were conducted with men aged 18–30, 31–50, and 51 years and above and another three with women stratified into the same age groups. We hypothesized that there may be differences in perspectives by stratum. The sub-county NTD focal point worked with community leaders to purposively select participants for the focus group discussions. Community leaders were asked by the focal point to contact individuals that resided in the community, present at the time of the study and most recent MDA, were over the age of 18, and were willing to come to a specified location and time the following day for an interview. The first 48 individuals to answer leaders’ calls and agree to participate were included in the study as participants. Community leaders informed participants about the location, day, and time of the interviews. In each sub-county, two trained public health interns from nearby health facilities moderated the focus group discussions, with one moderator assuming the role of note taker. The total number of participants in each focus group was eight. The focus group discussions were conducted in a private room, at either the railway dispensary or sub county health center. The venues were chosen based on the convenience for the participants. The discussions were conducted in KiSwahili, the national language of Kenya, and lasted for approximately 75 min. A topic guide was used to facilitate discussions.

#### Semi-structured interviews

In each sub-county, nine CHEWs and seven community leaders were purposively selected and interviewed. The county NTD and national LF focal points were also interviewed. Participants were identified and recruited by a sub-county NTD program official, and were 18 years of age and above and participated in the most recent MDA. In each sub-county, two trained public health interns from nearby health facilities conducted the interviews. The interviews took place in a private room at the closest health centre or in the participant’s home, and lasted for approximately 60 min. The interviews, conducted in KiSwahili, explored a range of topics related to CDDs and community experiences during MDA.

### Quality control

In order to ensure data quality and optimization, interview guides were translated from English to KiSwahili and KiSwahili to English in order to reconcile discrepancies and differences. One of the authors and a senior research assistant that spoke KiSwahili supervised all interviewers to ensure that interviewing and data capture techniques were adhered to. After interviews, interviewers reviewed and added to their notes, and uploaded recordings to the cloud. Interviewers transcribed recordings into English at the end of each day. The senior research assistant randomly selected two recordings and transcripts from each interviewer to ensure consistency between recording and transcription.

### Data analysis

Interviews with forty-eight focus group participants, eight CHEWs and six community leaders in each sub-county were digitally recorded and transcribed verbatim. Transcripts were translated to English, reviewed, and then manually coded. Content analysis of the data was conducted (Young Cho & Lee, 2014), which involved a process of reading, coding, and displaying data in order to develop a codebook. Members of the study team read a subset of transcripts and coded emerging themes, reviewed coding, and refined the codebook through an iterative process until consensus was reached between the authors. In order to reach consensus, analyses, codes and themes on the same text were compared to examine similarities and differences. Where there were differences, we actively re-read the text and discussed perspectives. This iterative process consisted of the integration of both deductive and inductive methods to identify predetermined and emergent themes (Young Cho & Lee, 2014). Predetermined themes were based on the study objectives, and included community engagement with CDDs, NTD program engagement with CDDs and CDD motivation and remuneration.

#### Ethical considerations

Informed consent was obtained from all study participants, including consent to digitally record interviews. This study was reviewed and approved by the Kenya Medical Research Institute Scientific and Ethics Review Unit, and a waiver was obtained from the Ethics Committee of Northwest and Central Switzerland, approval number 2018–00694.

## Results

A total of 10 themes emerged from the data (Table 1). They are classified into three groups: themes, sub-themes, and perspectives. Sub-themes and perspectives did not differ by age and gender.

**Table 1:** Perceptions of CDDs

Themes	Sub-Themes	Perspectives
Communication between CDDs and the community	CDDs hurriedly distribute drugs, resulting in little to no health education.	Adequate health education enables community members to understand LF as a disease with devastating outcomes. Once this is understood from CDDs, they are more likely to participate in MDA and ingest medicines.
Qualities of good CDDs	Trust is established between CDDs and communities when CDDs are nominated and selected by their community. CDDs that are perceived to behave well and have a good image are desirable.	When trust is established, CDD interactions with community members are positive. As a result, community members are able to trust information from CDDs about the importance of MDA and ingesting medicines. Conversely, when CDDs are not trusted, community members are hostile and refuse medicines. CDDs must be known and selected by their community in order to achieve their goals during MDA. CDDs that are not only selected by community members, but are also well mannered and respectful are embraced and trusted.
Community resistance toward CDDs	Community members’ distrust of CDDs often led to sharp resistance and negative treatment of CDDs.	Drug misconceptions stemming from cultural beliefs, political propaganda, and lack of awareness fuel resistance to MDA and CDDs in the community.
Community distrust of the federal government	Community members perceive that the government is motivated by a political agenda, and that MDA is the platform used to advance their harmful agenda.	Community members perceive MDA to be a way for the government of Kenya to enforce family planning. This misinformation results in a lack of participation in MDA.
CDD motivation	Community members perceive that CDDs are motivated when they feel appreciated by them.	When communities are receptive to the health information, look for CDDs to obtain the drugs, encourage their neighbors to accept the drugs, and provide water, airtime and food, CDDs often feel appreciated. Monetary incentives also serve as motivation for some CDDs to perform well, especially those who are unemployed. Nonetheless, the primary motivator for volunteering among CDDs is their commitment to serve the community.
Perception of MDA as a distribution strategy	Community members that perceive that drugs should be distributed in private facilities or hospitals often don’t participate in MDA and belong the higher income groups. On the other hand, those in the lower income groups embrace MDA.	CDDs find it challenging to reach those in higher income levels because they perceive that access to medicines should be from private doctors and hospitals in the event that they do fall sick. This perception may suggest that LF is a disease associated with poverty and that CDDs are not qualified to offer them medicines.
Timing of MDA	The period during which CDDs distributed medicines has an impact on coverage.	Some community members are frequently absent during MDA because of work, travel or school.
Community accountability during MDA	Community elders often help achieve treatment coverage through indirect supervision of community members and CDDs during MDA.	Community leaders serve as informal supervisors by addressing troublesome community members and by encouraging them to accept and ingest medicines.
CDD bias	CDDs purposively decide which individuals and groups would receive medicines, which impacts treatment coverage.	Community members believe that CDDs should offer drugs to everyone and not those they like.
Community understanding of CDD job responsibilities	CDDs are expected to engage in a variety of activities before, during and after MDA.	Directly observed treatment, health education prior to MDA, dosage assessment, and mop-up are all responsibilities of CDDs. Community members report forgetting to take the drugs when CDDs left the drugs, without implementing directly observed treatment.

*CDDs* Community drug distributors; *LF* Lymphatic filariasis; *MDA* Mass drug administration.

### Focus group discussions

Participants shared that LF is caused by mosquitoes, and manifests as swelling of legs, scrotum and breasts. They also expressed that LF can be controlled with drugs and using mosquito nets at home. The reasons cited for risk of LF included poor waster disposal, being near the ocean and the presence of mosquitoes. Only one focus group participant reported that worms caused LF. Perception of risk for LF was high among focus group participants. Acceptance of LF medication during the last MDA varied among the groups. There were some observed differences by age and gender among those that reporting having taken the drugs and those that did not.

### Semi-structured interviews

In each sub-county, five out of six community leaders reported incomplete primary school education, and all CHEWs had at least a secondary school degree. Most participants identified themselves as Muslim.

#### Communication with CDDs

Some participants indicated that lack of knowledge of LF and MDA made it difficult for them to accept CDDs and the medication. Mainly, they expressed a lack of effective communication with CDDs influenced their decision to accept medicines. In addition, CDDs are often in a hurry and don’t provide adequate explanations about medicines.

*Some have bad approach to the community. Some are in hurry and therefore don’t explain the usage of drug and why are people being given drugs.* (FGD, men, 30–50 years old)

*Some CDDs teach us in a way we don’t get to understand the message. Some people are slow learners.* (FGD, women, 50 years and above)

#### Qualities of a good CDD

When participants discussed qualities of a good CDD and how CDDs were selected, they consistently reported the importance of CDDs’ relationship with the community. Focus group discussions and interviews with CDDs and community leaders revealed that CDDs that were from the community or those that were well known among community members were trusted and accepted. Furthermore, participants described how a trusting relationship between the community and CDD made it less challenging for CDDs to gain access to the households to distribute medicines.

*The community give me positive feedback about them, they say that they are respectful and they interact well with them. Their relationship is good because they all come from the same community and have a good understanding with each other. The community is welcoming to them because they know each other.* (Elder, woman, 63 years old)

On the other hand, CDDs that are not selected by community members will not be given the opportunity to distribute medicines because they are not trusted.

*… We have never seen them in the community that’s why it’s hard for some to accept the drugs. I would trust someone from within the community more than strangers.* (FGD, women, 18–30 years old)

Participants mentioned that CDDs were chosen to volunteer for MDA based on their relationship with the community, work history, and ability to perform the job tasks (e.g. climbing tall buildings). Several participants suggested that CDDs that were well mannered, respectful, trustworthy, and had a good reputation were well received by the community. Participants also mentioned that patience was an important quality for CDDs to possess because they endured a great deal of resistance from the community.

*Like I told you, we don’t choose people if they have a bad image, unless that person has good relations with the community that’s when our work also becomes easier because already there is a connection. If a CDD is not respectful then there wouldn’t be a good relationship. (Elder, man, 54 years old)*

#### Community resistance toward CDDs

Over 20 interview participants expressed the view that CDDs endured harsh treatment from community members during drug distribution. Experiences of harsh treatment included being chased away, having doors slammed in their faces, and encountering insults.

*You know people can’t be the same, sometimes a CDD could knock at the door but they don’t open or when they open they become harsh, abusive and bang the door at your face. (Elder, woman, 64 years old)*

#### Community distrust of government

The most common drug misconceptions reported by participants included that drugs were not safe and intended for promoting family planning.

*Many people have lost trust with CDDs because some think its political, there is some registration that goes on before the MDA, people think it is connected to politics that there may be rigging of elections. Some believe it’s a way that the government is using to reduce the population, a way of family planning.* (FGD, women 50 years old and above)

*There is a challenge because you know there is the rich, poor and middle class. The rich have problems, they have the attitude that they are being given family planning drugs, not opening the doors or if they do, then they insult you.* (CHEW, two years of experience)

#### Perception of MDA as a distribution strategy

Participants also mentioned that some community members, primarily the higher class, did not accept the drugs because they prefer to be treated in a hospital or by their own private doctors.

*Some people chase the CDDs away saying they have their own private doctors that they can go to in case of any medication.* (Elder, man, 50 years old)

*It depends on the community, you find that some of these people that are in the middle and high-income level, feel that MDA is beneath them. They feel that they can access better services in private facilities, but you find that in the informal sectors and low income earners, they embrace the MDA in fact they ask for the medicines. So there is always this disparity between the high and low-income earners.* (CHEW, two years of experience)

#### CDD motivation

Elders and community members shared that they believed that CDDs were more motivated when they felt appreciated and supported by the community. They also felt that CDDs appreciated it when community members received them well, were receptive to the health information, and accepted the medication. Participants also shared the view that CDDs felt appreciated when community members looked for them to obtain the drugs, as well as encouraged their neighbours to accept the drugs. Several participants described instances when community members expressed gratitude and provided CDDs with resources such as water, food, and airtime.

*In some households they express their appreciation by providing water, milk or refreshments considering the fact that they are not provided these things during the MDA is something positive for them.* (Elder, man, 50 years old)

*Yes I feel they are rewarded and appreciated because we listen to what they have to tell us and accept what they have to offer. We also respect them for that.* (FGD, women 18–30 years old)

Community members and leaders indicated that extrinsic rewards, especially the monetary incentive, served as a significant motivator for CDDs.

*Some CDDs do the job as a source of income, so their motivation is the money they get after the job. So many of us are unemployed.* (FGD, women, 18–30 years old)

#### Community level accountability

Some participants suggested that community leaders served as informal supervisors. Community leaders accompanied CDDs in the field and ensured they performed their duties. Several participants also described how community leaders assisted CDDs when they faced resistance in the field. Community leaders were crucial in helping CDDs gain the trust of the community. Participants also discussed how community leaders supported CDDs by helping to dispel misconceptions about the drugs and assisting with difficult community members.

*Other people refuse the drugs when given by other CDDs unless they see me. When I show up they calm down and accept.* (Elder, woman, 64 years old)

*Incitement among the community members, some spreading negative news about the medicines that’s why village elders are involved to convince the community that the stories are not true. They believe in us even more than the sister or nurse accompanying us because they don’t have enough interaction with the community.* (Elder, man, 50 years old)

#### Timing of MDA

Focus group and interview participants discussed the issue of participants not being available during MDA. The reasons for community members’ unavailability during the MDA included work, school, and travel. This resulted in low coverage, especially when CDDs did not return to the household.

*The problem was time factor they want to distribute the drugs during the day and that is the time when most people are at work. Supposed they were doing it at night, when we are all at home in the evening or at night then all of use would have taken medicines.* (FGD, men, 30–50 years old)

#### CDD bias

For focus group participants, some CDDs are biased when distributing drugs so that community members are not offered the medicines.

*They should be able to give drugs to all not denying others.* (FGD, men 30–50 years old)

#### Community knowledge of CDD job responsibilities

The majority of participants mentioned that CDDs were expected to distribute drugs door to door, provide education on LF and the importance of drugs, and administer the proper dosage. Several participants expressed that CDDs were expected to perform directly observed treatment, without force, and to follow up with missed clients to ensure all households are met.

*Before the MDA, the CDDs come to inform us after which they distribute the drugs. After the MDA, I see the CDDs coming around to check on those who had missed out on the drugs.* (FGD, women, 18–30 years old)

Participants pointed out that some CDDs left the drugs for household members that are not around at the time of distribution or allowed household members to ingest the drugs later. Several focus group participants reported that some community members did not take the drugs because they forgot to take them later or they disposed of them instead.

*“You see some CDDs bring the medicine but do not wait to watch someone swallow. They just give and go so in that case they don’t swallow.* (FGD, women 30–50 years old)

Interviews with the national LF focal point and county NTD focal points revealed similar themes around barriers and facilitators of implementing successful MDA campaigns. First, the program’s limited financial resources for MDA lead to inadequate pay for CDDs, as well as high workload given the limited number of days allocated for MDA. In addition, treatment coverage is often affected by individuals’ strong cultural and religious beliefs around LF and the purpose of the medications. For example, Muslim husbands might prevent their wives from ingesting drugs because of their fear it may result in infertility. Furthermore, perceptions around the causes of LF are informed by cultural beliefs passed on from generation to generation, making it difficult for some communities to participate in MDA. Given Kenya’s political challenges over the last decade, many communities have a deep lack of trust for government programs and initiatives. The focal points agreed that more resources are needed to better educate communities about LF, gain the support of community leaders, develop additional fixed posts for distribution of medicines, improve supervision of CDDs, access hard-to-reach areas, and motivate CDDs to achieve their MDA targets.

## Discussion

The current study systematically examines perceptions of CDDs from the perspectives of multiple stakeholders in coastal Kenya. The use of qualitative research methods is effective in exploring the meanings of social phenomena experienced directly by the individual, resulting in the collection of detailed, rich information [17]. The findings suggest that gaining community acceptance and trust, as well as being selected from their own communities, impact CDDs’ ability to reach household targets, as well as end users’ decision to ingest medicines. In Cameroon and Uganda, CDD connectedness to the community was also emphasized among study participants as a major deciding factor on whether or not community members received CDDs and accepted the medicines [12, 18]. Additionally, CDDs that are well-mannered, patient, effective communicators, and knowledgeable are well received by the community and perceived to perform well on the job [10]. In our study, CDDs were not accepted or trusted due to poor community awareness of MDA, cultural and religious beliefs, and political propaganda. These findings are consistent with those of Ames et al., 2019 and Ahorlu et al., 2018 where community trust and selection of CDDs positively impacted treatment coverage [14, 19]. Participants also suggested that signs of appreciation from community members, such as provision of water and warm reception encouraged CDDs, whereas encountering hostile community members often discouraged them. Another relevant finding was around community distrust of the government. Participants shared that they believe MDA is the government’s attempt to promote family planning. Odour has studied the integrity in public health sector service delivery among the population in Busia County, Kenya. Findings indicated that 68% of the respondents were of the opinion that corruption in the public health sector was a very serious problem. Respondents reported corruption practices, such as harassment from providers, extortion, absenteeism of providers and informal payments required for treatment [20]. Corrupt practices within the health system may fuel community distrust of the NTD program, including CDDs.

The negative perception of MDA as a distribution strategy among higher income individuals was another interesting finding. These individuals feel that they have no symptoms, and therefore, do not have a need to ingest medicines. They also do not find it appropriate to ingest medicines provided by CDDs given that they have access to private doctors and hospitals. However, individuals in the lower income classes embrace MDA. This suggests that higher income individuals may associate LF with poverty, do not trust the NTD program, and have limited awareness of LF and the importance of MDA. Lymphatic filariasis often affects the socially marginalized and poor due to lack of use or access to mosquito nets. In addition, morbidity and disability due to LF significantly reduce economic output and increase poverty [21]. The lack of education and awareness, which may suggest a communication challenge with CDDs, around MDA and LF has been well documented. A study conducted by York et al., 2014 found that lack of awareness and education around LF was observed in both community members and CDDs [22]. The findings also suggest that the timing of MDA affects CDDs’ ability to distribute drugs to community members and to reach targets. Brieger 2000 found that community members did not respect the volunteers’ time, approaching them at any time of the day [23]. Furthermore, they were often absent during distribution, requiring the volunteer to visit again. This is because most people may be at work or traveling. Chami and colleagues in Uganda have documented CDD bias during MDA. They found that CDDs were more likely to deliver drugs to individuals who trusted them for health advice and were influential in the community, which allows them to maintain their own influential status [24]. This means that less privileged individuals were less likely to be offered treatment. Social bias from CDDs during MDA may also be due to lack of sufficient monetary incentives, distance between households, and limited resources. Finally, the role of community leaders as informal supervisors that address myths about MDA and support CDDs with drug distribution serves as an important facilitator for achieving MDA targets.

The findings of this study suggest that, even if medicines are available, community members may not accept them because of distrust of the government, poor interactions and communication with and between CDDs and communities, and lack of knowledge and awareness of MDA and LF. Social bias from CDDs and timing of MDA affect some individuals’ ability to access medicines during MDA. Medicines must be located within reasonable reach of the people who should benefit from it. CDDs must also have the capacity, motivation, time and resources to distribute medicines relation to the size of the target population. In order for the NTD program in Kenya to achieve epidemiologic coverage, the different dimensions of effective coverage must be addressed. Table 2 below shows specific proposed solutions for improving MDA by theme and sub-theme. At the national level, the NTD program can enact policies that ensure that MDA is held at fixed posts, and in public spaces to increase access to medicines for all individuals. Furthermore, the roles of community elders can be clearly defined to reflect supervision, positive reinforcement and health education support during MDA. This will ensure that all elders across communities help to improve MDA and support CDDs in a uniform, systematic and impactful way. With a growing population and pressures to meet targets, CDDs may be required to distribute medicines to communities that did not select them; however, the local NTD program can actively work with community leaders to recruit CDDs on an ongoing basis and place them on standby. Pre-MDA activities at the national and local levels can include digitally based messaging that assures community members of integrity in the health system, government and service providers. CDD motivation can be sustained through supportive supervision and adequate training, incentives and resources. As concluded by Krentel and colleagues, implementing and testing solutions for improving CDD motivation should be done so through the health system for sustainability, and leverage novel approaches such as digital technologies to effectively engage communities [15].

**Table 2:** Solutions to improve MDA

Themes	Sub-themes	Solutions
Communication between CDDs and the community	CDDs hurriedly distribute drugs, resulting in little to no health education.	• CDDs can be given an extra day for distribution or sensitization in order to provide adequate health education for communities. • CDD trainings can include modules that emphasize the importance of and justification for providing health education to households before administering medicines.
Qualities of good CDDs	Trust is established between CDDs and communities when CDDs are nominated and selected by their community. CDDs that are perceived to behave well and have a good image are desirable.	• Prior to the implementation of MDA, community leaders should be provided with a roster that lists all CDDs assigned to their community, and ensure that CDDs are known to and accepted by the community. This can be done during standing community meetings. • CDD trainings can include modules on expectations of community members for good behavior and image, and how to achieve that.
Community resistance toward CDDs	Community members’ distrust of CDDs often led to sharp resistance and negative treatment of CDDs.	• Prior to MDA implementation, community leaders can hold a meeting or forum introducing CDDs and explaining their roles. During this meeting leaders can emphasize the importance of accepting MDA and medicines • Increase CDDs’ knowledge of LF through adequate training before MDA • Provide CDDs with the job resources, aids and time they need to adequately conduct social mobilization activities before MDA • Community leaders and key popular figures can show community members that they have ingested medicines.
Community distrust of the federal government	Community members perceive that the government is motivated by a political agenda, and that MDA is the platform used to advance their harmful agenda.	• Government leaders at all levels can join community meetings and record videos for various social media platforms to promote MDA and the importance of ingesting medicines. • Government leaders can collaborate with celebrities to use digital media to build trust and assure the public that MDA and medicines are safe and important.
CDD motivation	Community members perceive that CDDs are motivated when they feel appreciated by them.	• Communities can pool resources like water and food, and provide it to CDDs during MDA. • During meetings with community members, leaders can encourage them to embrace CDDs, show them appreciation and help them where they can • The NTD program can ensure that program staff and supervisors are always within walking distance of CDDs to reduce out of pocket expenses on airtime and transport.
Perception of MDA as a distribution strategy	Community members that perceive that drugs should be distributed in private facilities or hospitals often don’t participate in MDA and belong the higher income groups. On the other hand, those in the lower income groups embrace MDA.	• The NTD program can offer those in higher income groups the option of obtaining their medicines from health centers or their own doctors.
Timing of MDA	The period during which CDDs distributed medicines has an impact on coverage.	• The NTD program can explore alternative dates for MDA, and ensure that CDDs have adequate time and resources to conduct mop-up. • The NTD program can explore distribution of drugs in workplaces, health centers and other fixed posts.
Community accountability during MDA	Community elders often help achieve treatment coverage through indirect supervision of community members and CDDs during MDA.	• Community leaders can serve as informal supervisors during MDA by conducting random checks on households and CDDs.
CDD bias	CDDs purposively decide which individuals and groups would receive medicines, which impacts treatment coverage.	• CDD trainings can include modules that emphasize the negative impact and consequences of overlooking certain individuals. • Supervisors can closely monitor the progress of CDDs during MDA, and ensure that daily targets are being met. Supervisor trainings should include modules of effective and supportive supervision of CDDs to reduce bias.
Community understanding of CDD job responsibilities	CDDs are expected to engage in a variety of activities before, during and after MDA.	• CDD trainings should emphasize the importance of directly observed treatment. • Community meetings held prior to MDA should include discussions around the responsibilities of CDDs.

*CDDs* Community drug distributors; *LF* Lymphatic filariasis; *MDA* Mass drug administration; *NTDs* Neglected tropical diseases.

The current study highlights not only the barriers to the successful implementation of MDA, but also the costs that CDDs bear in order to meet NTD program demands. It also highlights the importance of the careful choice of CDDs in the fight against LF, as well as the need for more detailed research. Future studies can include comparisons across CDDs in areas with high coverage and low coverage, and more in-depth exploration on CDDs’ job-related experiences and their professional quality of life.

One of the main limitations of the study is the purposive selection of study participants by the sub-county NTD program. In preparing for the study, we were expected to rely on the NTD program’s community health liaison to identify participants, which may have resulted in a biased sample and respondents providing interviewers with socially desirable responses. The study could have been strengthened by conducting separate focus groups for community members that did not participate in the previous MDA and those that did.

## Conclusions

The effective engagement of communities and CDDs in the fight against LF is critical to the achievement of national and international elimination goals. Given the introduction of new global targets and drug therapies, there are added pressures on NTD programs to deliver medicines to at-risk groups efficiently and effectively. Additional treatment rounds along with its associated enormous financial investments by donors may not be sustainable, threatening the effectiveness of NTD programs to efficiently and effectively deliver treatment to at-risk groups. In order to address these challenges, changes at both the macro and micro levels must take place. If the NTD programme in Kenya continues to achieve low coverage, they will have to continue to treat areas like Kaloleni and Mvita sub-counties, and possibly experience delays in achieving WHO targets. This also means that the country program will require additional financial, human, and biomedical investments in order to ensure that various stakeholders are effectively engaged. If country NTD programs are going to be successful in maximizing resources, sustaining the gains made, and achieving elimination goals, it is imperative to tackle MDA challenges at all levels of the health system.

## Data Availability

The dataset supporting the conclusions of this article is available on request from the lead author.

## Abbreviations

CDD: Community drug distributor
CHEW: Community health extension worker
LF: Lymphatic filariasis
MDA: Mass Drug Administration
NTDs: Neglected tropical diseases
WHO: World Health Organization
